# Assessing the efficacy of jet dispenser versus direct syringe injection for calcium hydroxide paste placement in artificial root canals

**DOI:** 10.1038/s41598-023-46683-y

**Published:** 2023-11-09

**Authors:** Youngwook Song, Hwichan Ham, WooCheol Lee, Ryan Jin Young Kim

**Affiliations:** 1 School of Dentistry, Seoul National University, 101 Daehak-ro, Jongno-gu, Seoul, Republic of Korea; 2 Department of Aerospace Engineering, Seoul National University, 1 Gwanak-ro, Gwanak-gu, Seoul, Republic of Korea; 3 Department of Conservative Dentistry, Dental Research Institute, School of Dentistry, Seoul National University, 101 Daehak-ro, Jongno-gu, Seoul, Republic of Korea; 4 Department of Dental Science, Dental Research Institute, School of Dentistry, Seoul National University, 101 Daehak-ro, Jongno-gu, Seoul, Republic of Korea

**Keywords:** Dental equipment, Endodontic instruments

## Abstract

This study aimed to evaluate the efficacy of a novel jet dispenser (JD) for filling simulated straight and curved canals with calcium hydroxide (Ca(OH)_2_) paste by comparing the level of intracanal medicament with traditional direct syringe delivery. The Ca(OH)_2_ paste was delivered into the canals using either a direct syringe with binding tip and unbinding tip or a JD. The analysis of the ratio of the canal filled area (RCFA) in the coronal, middle, and apical thirds was conducted using the Kruskal–Wallis and Mann–Whitney *U* tests (α = 0.05). The JD resulted in a significantly higher RCFA of 1.00 in the middle and apical thirds in both the straight and curved canals than the direct syringe method (*p* < 0.001). Voids were frequently observed in the direct syringe group, whereas JD showed no voids within the filled area. In the direct syringe groups, the apical area was more readily filled in both canal types when the tip was bound (*p* < 0.001). The JD was found to be more effective in filling the entire canal space than the binding and unbinding conditions of the direct injection method, particularly in the apical area.

## Introduction

The key to a successful root canal treatment is the complete elimination of pathogenic microorganisms that adhere to the root canal walls or become trapped in the dentinal tubules^1^. The complex anatomy of root canals, including the isthmuses, accessory canals, and apical deltas,makes it difficult to completely eradicate bacteria from infected canals^2–4^. Therefore, the use of intracanal medicament between appointments has been recommended to reduce the number of residual bacteria^5,6^.

Although numerous materials have been tested over decades, calcium hydroxide (Ca(OH)_2_) is considered the gold standard intracanal medicament in endodontics^7–9^. Its high pH 12.6 exerts an antibacterial effect by releasing hydroxyl ions in an aqueous environment, which produce highly oxidizing free radicals that directly contact and kill the bacteria^10^. In addition, Ca(OH)_2_ has tissue-dissolving properties and low water solubility, which allows it to act as a chemophysical barrier to reduce re-contamination by bacteria^11^. To achieve these effects, the medicament must fill the entire length of the root canal and contact the tissue in areas inaccessible by mechanical instrumentation^12^. However, it is challenging, even for endodontists, to completely fill the medicament into the root canal without underfilling or extruding beyond the apex. Direct injection of medicament using a syringe containing the paste is the commonly employed method despite the difficulty in controlling the amount of medicament that is injected^13^.

In the medical field, a jet dispenser (JD), also known as a transdermal drug delivery system, has recently been developed and made commercially available^14^. These devices offer rapid and precise delivery of drugs per injection into the subdermal area while minimizing tissue destruction, thereby reducing pain and ecchymosis^15^. JD is characterized by its ability to penetrate the dermal barrier without the use of a needle. The precise drug delivery capability of the JD has the potential to be a valuable tool in dentistry, particularly for the precise delivery of intracanal medicaments into the root canal. However, no study has evaluated the potential applicability of the JD for delivering Ca(OH)_2_ in the root canal. Therefore, this study aimed to assess the efficacy of this innovative drug delivery device for filling simulated canals with Ca(OH)_2_ by comparing the level of intracanal medicament placement with the JD to that obtained using the traditional direct syringe injection method. The null hypothesis was that there would be no significant difference in the levels of Ca(OH)_2_ placement between the two injection techniques (JD vs. direct syringe).

## Methods

### Preparation of the root canal model

Computer-aided design software (Rhino 3D; Robert McNeel and Associates, Seattle, WA, USA) was used to create block models of the straight and curved root canals. All canals were designed to have a taper of 0.06, with an apical diameter of 0.30 mm, and a total length of 14 mm. The straight canal model featured a straight-line canal, whereas the curved canal model had a 30-degree curvature from the middle third toward the apex. Each block had a cylindrical hole (2 mm in height and diameter) at the apex. Using a digital light processing three-dimensional (3D) printer (IMC; Carima, Seoul, Korea), 36 blocks were printed for each type of root canal model at a layer thickness of 50 μm with a photopolymerizable resin (Dental Model; Carima). After printing, the blocks were washed in isopropyl alcohol for 10 min (Form Wash; Formlabs, Somerville, MA, USA), followed by additional rinsing with isopropyl alcohol through the canals and post-curing for 30 min (Form Cure; Formlabs).

To standardize the canal size, the canals were shaped using the ProTaper Next X2 (0.25 mm tip with 6% taper) and X3 (0.30 mm tip with 7% taper) Ni–Ti files (Dentsply Maillefer, Ballaigues, Switzerland) with the X-Smart Plus motor (Dentsply Maillefer) in the “ProTaper Next” mode (300 rpm and 2.5 Ncm torque). Following each instrumentation, the canals were thoroughly irrigated with 2 mL of saline and dried using paper points.

### Application of intracanal medicament

A water-based pre-mixed Ca(OH)_2_ paste with barium sulfate (Any-Paste, Mediclus, Cheongju, Korea) was applied to the prepared canals using either the conventional direct syringe injection or JD method.

For the direct syringe injection method, 24 blocks of each canal type were subdivided into two groups based on the initial position of the delivery tip. In one subgroup (n = 12), the tip was initially bound to the canal, whereas in the other subgroup (n = 12), the tip was retracted to avoid binding to the canal during the initial paste placement. In both groups, a 24-gauge tip was fitted to a syringe containing the pre-mixed paste, and the plunger was pressed to inject the paste into the canal while carefully withdrawing it coronally until the paste was flush with the orifice of the block.

**Figure 1:**
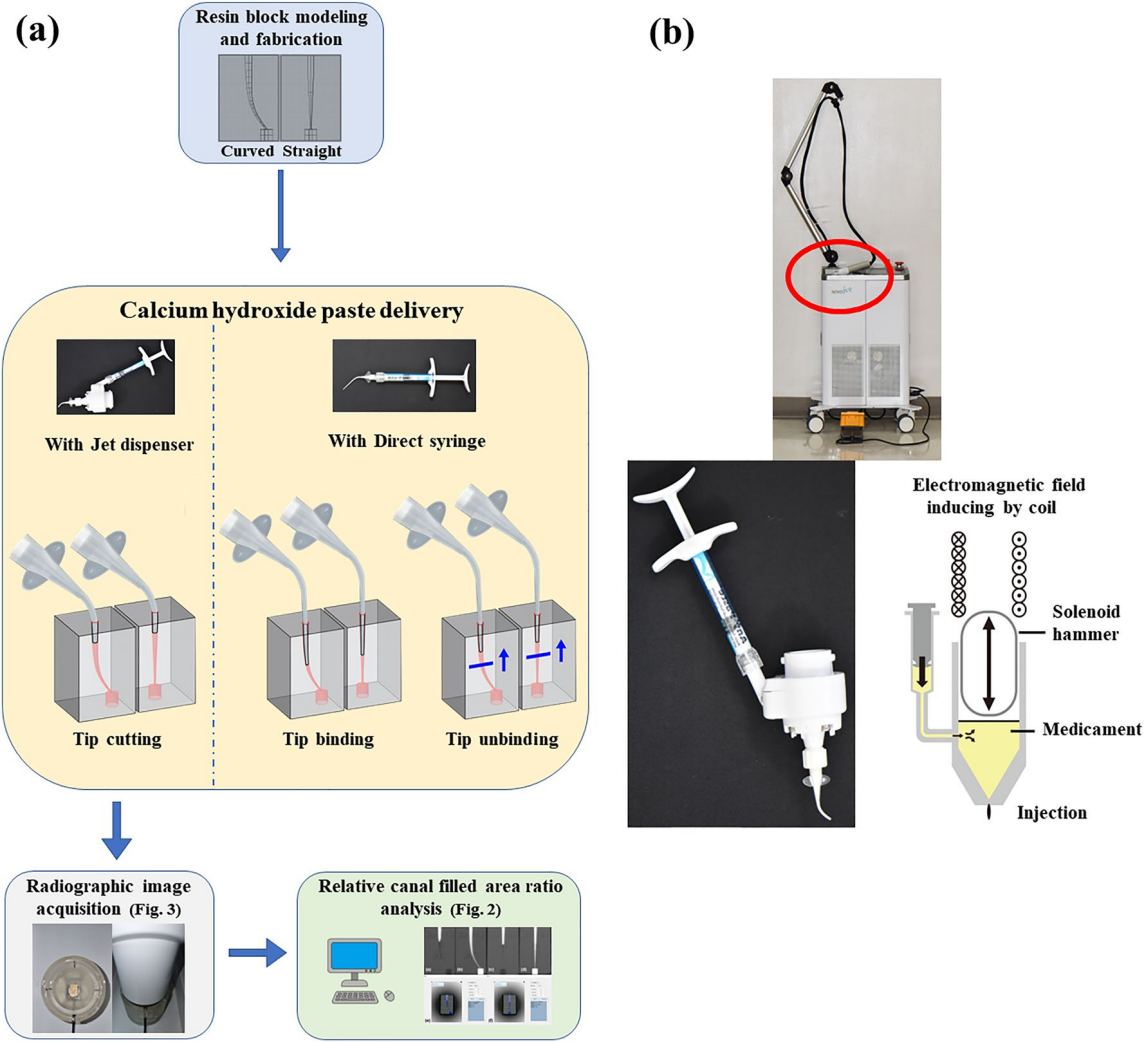
(**a**) Schematic diagram of the experiment. (**b**) Jet dispenser utilized in the experiment. The red circle in the jet dispenser is enlarged below, displaying the pre-mixed syringe fitted into the electromagnetic jet device, along with a schematic diagram.

**Figure 2:**
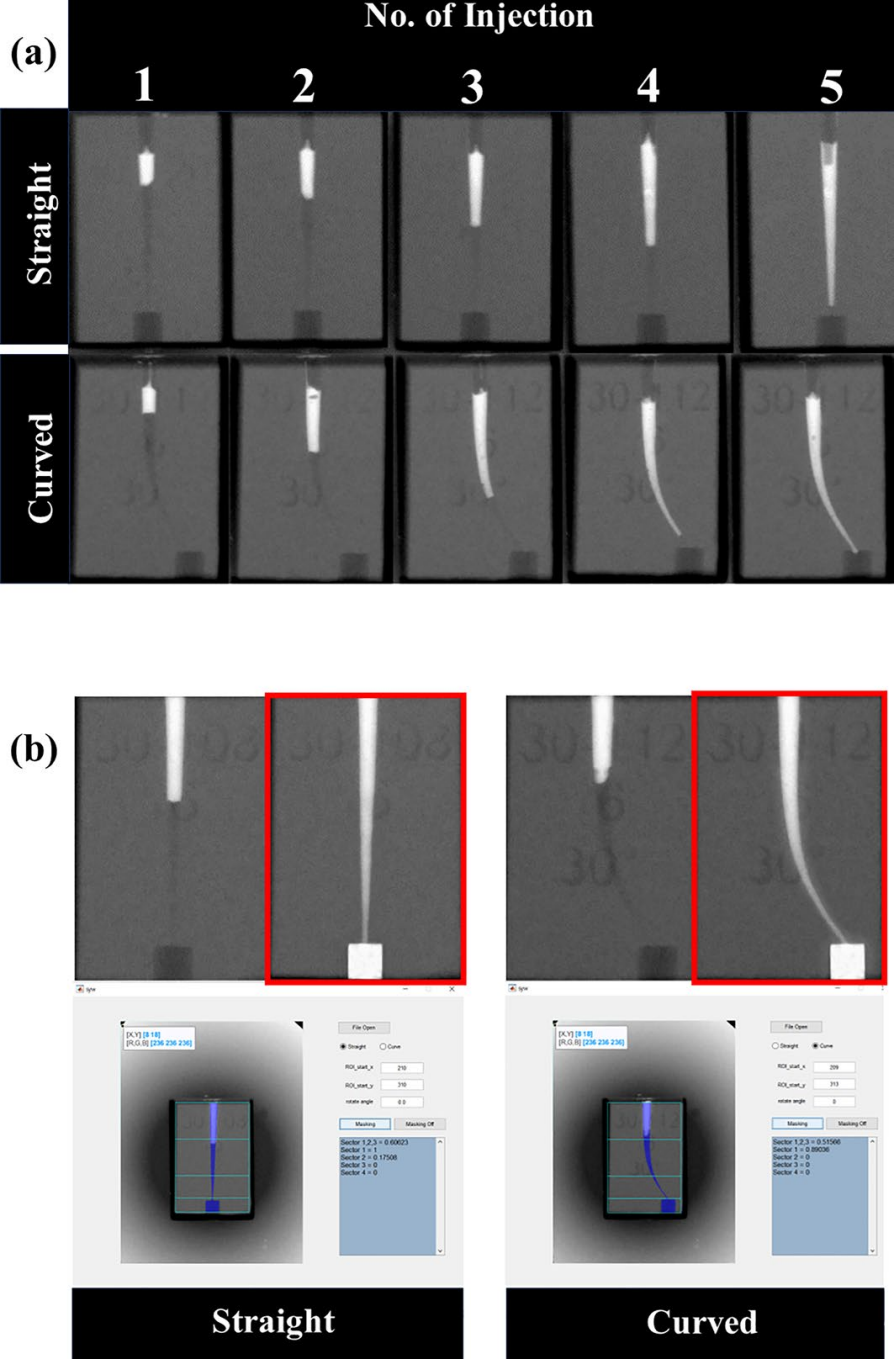
(**a**) Radiographic images displaying the distribution of Ca(OH)_2_ after a series of injections (injection 1 to 5) in both straight and curved canals using jet dispensing in the pilot study. (**b**) Radiographic image analysis conducted using the MATLAB program. The filled space was analyzed by comparing it with a reference image denoted by the red box, representing complete filling.

For the JD method, 12 blocks of each canal type were used. The JD method used in this experiment (Novojet, Bazbiomedic, Seoul, Korea) operates according to the following principle: an electric current applied to the coil generates an electromagnetic force that propels the hammer forward. Upon collision with the membrane, momentum is transferred to the drug, causing it to be sprayed from the nozzle onto the target area (Fig. 1). In our pilot study, the delivered amount of Ca(OH)_2_ was found to be proportional to the number of injections (Fig. 2). For the delivery of Ca(OH)_2_ intracanal medicament using the JD, five injections were administered to each canal. To ensure proper application, a 24-gauge tip was cut to a diameter of 1 mm to fit the coronal orifices of the blocks. All procedures were repeated for both straight and curved canal blocks by a single endodontist.

### Evaluation of distribution of intracanal medicament

**Figure 3:**
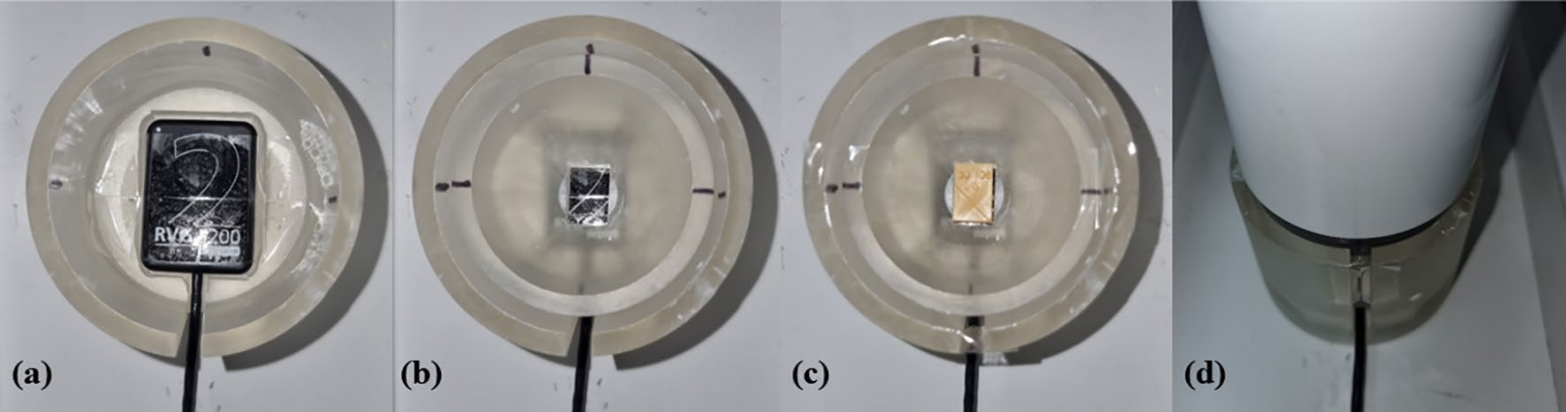
Customized 3D-printed jig for standardized radiographic imaging. (**a**) X-ray sensor held with the first part of the jig. (**b**) Second part is inserted to secure the position of the X-ray sensor. (**c**) A resin block is inserted into the second part of the jig. (**d**) X-ray cone is in place.

To ensure precise and consistent image acquisition, a custom-made jig was created using a 3D printer (IMC, Carima) (Fig. 3). The jig was designed in two parts: the first part was to hold the X-ray sensor securely, and the second part was to hold the canal blocks and X-ray cone in place, maintaining a stable and repeatable position throughout the imaging process. This allowed the X-ray beam to pass through the blocks in a parallel manner, thereby minimizing image distortion. The jig also maintained a consistent distance between the X-ray source and the blocks, ensuring an accurate comparison of the resulting images. After positioning the blocks and the X-ray cone using the jig, radiographic images of the blocks were acquired using a digital X-ray imaging system (RVG 5200, Carestream Dental, Atlanta, GA, USA) and saved in JPG format.

The images were imported into MATLAB software (MathWorks, Natick, MA, USA) to calculate the ratio of canal filled area (RCFA) using a baseline image created with Adobe Photoshop CS6 (Adobe Systems Inc., San Jose, CA, USA) as a reference, which simulated a root canal space fully filled with Ca(OH)_2_ (Fig. 2). For evaluation of the quality and distribution of Ca(OH)_2_ delivery within different regions of the canal; the areas were divided into three specific sections: “coronal 1/3” covered the area from the orifice to 5 mm below it; “middle 1/3” covered the area from 5 mm below the orifice to 10 mm below it; and “apical 1/3” covered the area from 10 mm below the orifice down to the apex. The RCFA in the preformed cylindrical space, that is, the amount of Ca(OH)_2_ extruded beyond the apex, was also evaluated.

### Statistical analysis

Data distribution was assessed for normality using the Shapiro–Wilk test, while the homogeneity of variances was tested using the Levene’s test. If the data did not exhibit a normal distribution, non-parametric tests, including Kruskal–Wallis and Mann–Whitney *U* test, were conducted using Statistical Package for the Social Sciences software, version 25 (IBM, Armonk, NY, USA) to compare the RCFA of Ca(OH)_2_ between the delivery methods at a significance level of 0.05.

## Results

**Figure 4:**
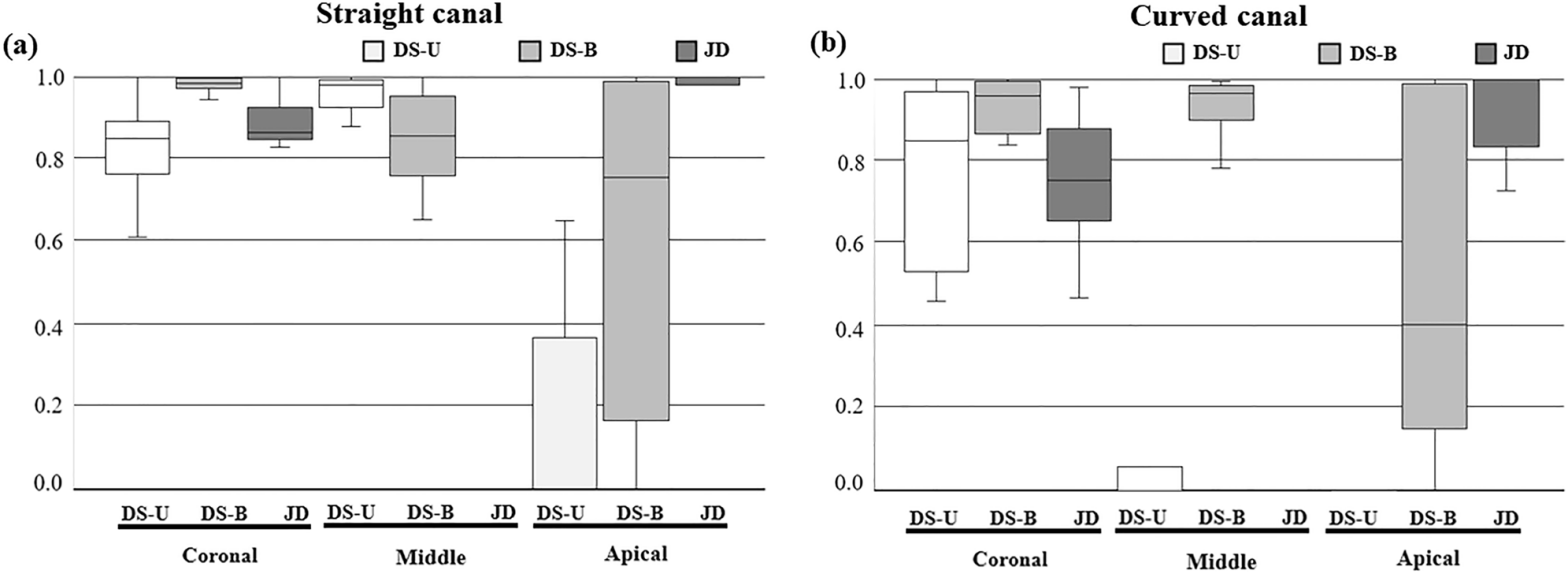
Box plot graph of ratio of canal filled area of (**a**) straight canal and (**b**) curved canal. *DS-U* direct syringe unbinding, *DS-B* direct syringe binding, *JD* jet dispenser.

The RCFA of Ca(OH)_2_ by the different groups in the coronal, middle, and apical thirds are shown in Table 1 and Fig. 4.

**Table 1:** Ratio of canal filled area by canal type and injection method.

	Coronal 1/3	Middle 1/3	Apical 1/3	Overfilling	Total
Straight canal					
DS-U	0.85B [0.77–0.89]	0.98B [0.93–0.99]	0.00C [0.00–0.37]	0.00A [0.00–0.00]	0.79B [0.73–0.84]
DS-B	0.99A [0.97–1.00]	0.86B [0.76–0.95]	0.76B [0.17–0.99]	0.00A [0.00–0.04]	0.91A [0.83–0.97]
JD	0.87B [0.83–0.93]	1.00A [1.00–1.00]	1.00A [0.98–1.00]	0.14B [0.00–0.39]	0.93A [0.91–0.96]
*χ*^2^	14.32	15.78	18.05	8.74	16.64
*p*	0.001	< 0.001	< 0.001	0.013	< 0.001
Curved canal					
DS-U	0.85B [0.53–0.97]	0.00C [0.00–0.00]	0.00B [0.00–0.00]	0.00A [0.00–0.00]	0.50B [0.31–0.58]
DS-B	0.96A [0.87–0.99]	0.97B [0.90–0.99]	0.41B [0.15–0.99]	0.00A [0.00–0.01]	0.90A [0.86–0.94]
JD	0.76B [0.66–0.88]	1.00A [1.00–1.00]	1.00A [0.84–1.00]	0.01B [0.00–0.10]	0.84B [0.77–0.93]
*χ*^2^	7.87	29.21	25.60	10.28	22.27
*P*	0.02	< 0.001	< 0.001	0.006	< 0.001

*χ*^2^, Chi square-value; p, p-value.

Interquartile ranges (first quartile, third quartile) are in parentheses.

Different capital letters indicate statistically significant difference between groups within the same area of interest (Kruskal–Wallis test, *P* < 0.05).

‘Ratio of canal filled area’ is the relative ratio of the filled area divided by the total area of simulated canal.

*DS-U* direct syringe unbinding, *DS-B* direct syringe binding, *JD* jet dispenser.

**Figure 5:**
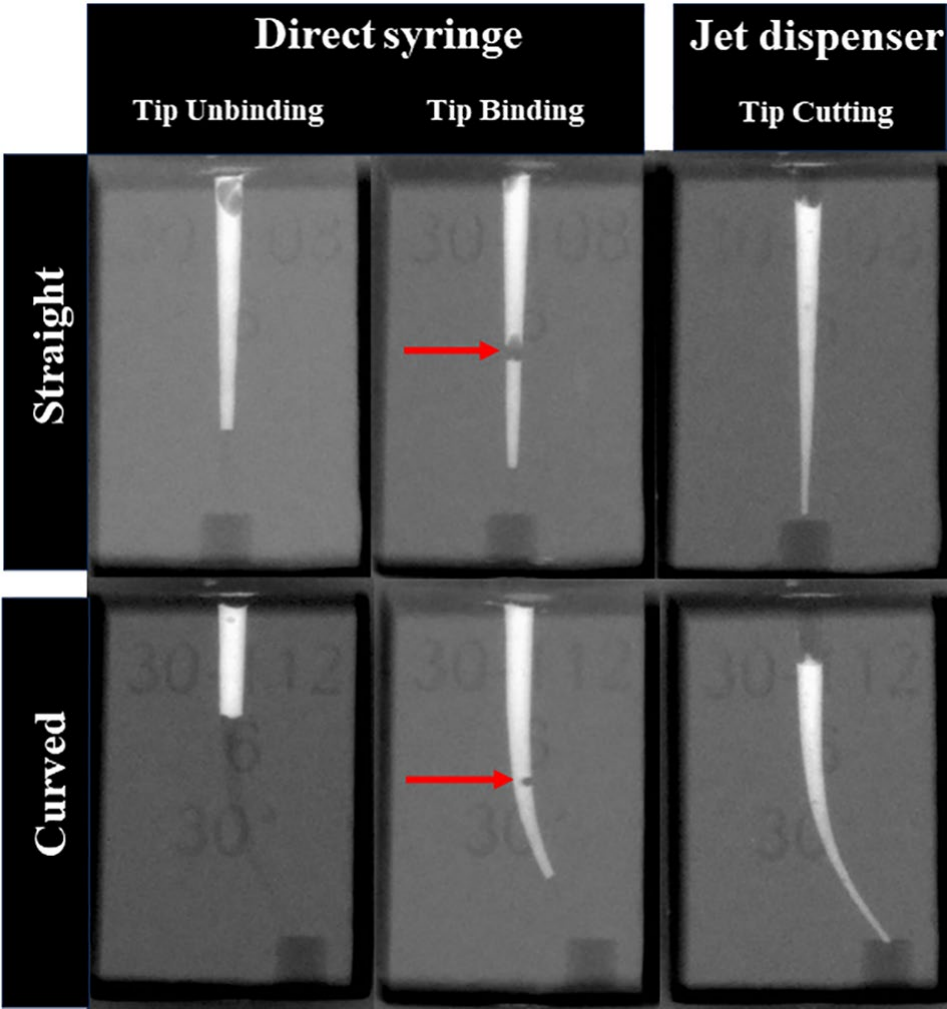
Representative sample image of each group. Red arrow indicates void inclusion.

JD resulted in a significantly higher RCFA of 1.00 in the middle and apical thirds of the canal than that of the direct syringe methods in both straight and curved canals (*p* < 0.001). In the direct syringe method, the median RCFA at the apical third was higher when the syringe was bound to the canal during initial placement. This difference was more significant in straight canals than in curved canals. However, the median RCFA was 0 in both straight and curved canals when the syringe was unbound. In the middle third, the unbound direct syringe method was canal type-dependent, with a median RCFA of 0 in the curved canal and 0.98 in the straight canal (*p* < 0.001). JD resulted in no voids within the filled area, whereas voids within the filled area were frequently observed in the direct syringe groups (Fig. 5). The occurrence of overfilling was significantly higher in the JD group than in the direct syringe groups, regardless of tip binding (*p* < 0.05).

## Discussion

To the best of our knowledge, this study represents the first evaluation of JD for the delivery of Ca(OH)_2_ intracanal medicament. The findings of this study demonstrated that JD performed better than the direct syringe delivery method in filling the canals, regardless of the canal type. Therefore, the null hypothesis was rejected, indicating that the JD may offer benefits over conventional delivery methods in terms of enhanced efficacy in filling canals.

For effective treatment, it is important that intracanal medicaments make contact with the maximum possible area of the canal space^12,16,22^. However, achieving an accurate length control using traditional delivery methods is technically challenging^17^. Clinicians often favor the direct injection technique because it is relatively straightforward and requires only a medicament syringe tip. Nevertheless, both underfilling and overfilling of intracanal medicaments can jeopardize the success of endodontic treatment.

A recent review of previous studies was unable to establish a definitive protocol for achieving optimal placement of Ca(OH)_2_ while preventing extrusion^18^. Generally, pre-mixed Ca(OH)_2_ syringes contain manuals that lack specific instructions on the delivery techniques. Instead, the manuals provide general guidance, such as “carefully filling the canal with the paste by pushing the syringe plunger while withdrawing the tip slowly.” The manual for certain products warns against overfilling and emphasizes the need for a radiograph if the medicament is not visible at the orifice 2 s after injection to confirm any extrusion^19^. However, the instructions provided in the manuals are vague and unclear, making it challenging for clinicians to achieve optimal placement of Ca(OH)_2_ without extrusion. Consequently, intracanal medicaments are often underfilled unless inadvertent overfilling occurs beyond the apex. Therefore, the direct syringe delivery technique relies heavily on the subjective judgment of the clinician, implying that the success of the procedure greatly depends on the skill and experience of the clinician.

The direct syringe technique was found to be inadequate for delivering medicaments to the desired location, especially in the apical third area, and is, therefore, not considered an ideal method for practical use. Even when the syringe tip was tightly bound to the canal during the initial placement, the direct syringe technique was still ineffective in filling the apical area of the curved canal. However, it was possible to inject an appropriate amount into the entire length using the JD, regardless of the curvature of the root canal, owing to its controllable amount per injection. In addition, the JD enabled even and adequate filling of the medicament without requiring technical expertise or subjective judgment from the clinician. However, in some samples, the JD resulted in extrusion beyond the apex, although not in significant amounts. This was expected because the direct syringe method could not even fill the entire apical area. Owing to the high pH of Ca(OH)_2_, extrusion should be avoided whenever possible to prevent complications such as transient-to-permanent numbness, chemical burns, abscesses, or sinus damage^20,21^. However, the limited amount of medicament delivered per injection in the JD reduces the risk of critical results, even in cases of apical extrusion.

The delivery of intracanal medicaments in a curved canal poses a challenge. The current injection method has limitations in effectively injecting medicaments into a minimally prepared curved canal. Studies have suggested that widening the canal up to a file size of #40 may be necessary for successful injection^22^. However, canal widening can compromise the structural integrity of the teeth^23,24^. Compared with the direct syringe method, specialized instruments, such as the Lentulo spiral, offer easier control of the length of the medicament delivered into the root canal system^25^. However, previous studies have reported that medicaments delivered using the Lentulo spiral have many porosities owing to the delivery mechanism, which spreads the drug to the canal wall^26^. To ensure the safety and effectiveness of treatment, using Lentulo spirals in curved canals is generally not recommended because of the increased risk of instrument fracture^27,28^. If the instrument is separated in the canal, it can significantly affect the tooth prognosis.

The potential risk of rotary instrument separation during medicament placement can be eliminated using a JD while ensuring effective filling of the entire length of the root canal system. The JD not only performs better in terms of filling level but also provides higher-quality medicament placement compared with direct syringe methods. This is evident from the absence of voids in the paste injected by the JD, whereas direct syringe methods often result in void formation, which might have occurred while withdrawing the syringe during placement.

In the field of endodontics, efforts to discover an ideal method of administering intracanal medicaments have not progressed significantly since the early 2000s. Recent studies have shifted their focus to techniques for removing the injected Ca(OH)_2_ for a hermetic apical seal. It appears that we have reached the limits of finding an ideal method, which is why a novel device capable of injecting accurate doses of drugs was introduced in this study. The original purpose of JD was to deliver medications with low viscosity through epidermal injection. However, when the 24-gauge tip of the syringe was attached to inject Ca(OH)_2_, the significant increase in the viscosity of the medicament made it challenging to control the amount of drug discharged per injection. In this study, the tip diameter was increased by cutting it, resulting in a higher output and enabling a precise release of Ca(OH)_2_. During the injection, the tip was positioned at the coronal orifice without the need for withdrawal, as required in direct syringe delivery. This approach not only resulted in consistent delivery but also helped avoid void formation (Fig. 5).

The limitations of this in vitro study include the use of only one type of JD with a particular level and speed of medicament release. Additionally, the study focused exclusively on a single paste, not addressing potential variations in viscosity that might have influenced the outcomes. Furthermore, the tests were performed using resin blocks with a single canal configuration, which may not fully reflect the complexity of natural teeth. Future research should adopt a three-dimensional evaluation and explore the applicability of the JD for delivering intracanal medicaments across diverse canal configurations, encompassing varying shapes and sizes. As an in vitro study, the findings might differ in an in vivo setting where factors such as positive apical pressure of periodontal tissues and blood supply may come into play. Further research is required to validate these findings and determine the optimal JD parameters for different clinical scenarios. Therefore, caution should be exercised when interpreting the results of this study, and the conclusions should be viewed as preliminary until confirmed through more extensive research. Nevertheless, the results of this study provide a general overview of the potential use of JD for delivering intracanal medicaments.

## Conclusion

JD exhibited potential for use in delivering Ca(OH)_2_ intracanal medicament paste into the canal space. JD was more effective in filling the entire canal space than the binding and unbinding conditions of the direct injection method, particularly in the apical third area.

## Data Availability

The datasets generated during the current study are available from the corresponding author on reasonable request.
